# Using Collagen Peptides From the Skin of Monkfish (*Lophius litulon*) to Ameliorate Kidney Damage in High-Fat Diet Fed Mice by Regulating the Nrf2 Pathway and NLRP3 Signaling

**DOI:** 10.3389/fnut.2022.798708

**Published:** 2022-02-10

**Authors:** Bingtao Miao, Jiawen Zheng, Guoping Zheng, Xiaoxiao Tian, Wen Zhang, Falei Yuan, Zuisu Yang

**Affiliations:** ^1^Zhejiang Provincial Engineering Technology Research Center of Marine Biomedical Products, School of Food and Pharmacy, Zhejiang Ocean University, Zhoushan, China; ^2^Zhoushan Institute for Food and Drug Control, Zhoushan, China

**Keywords:** collagen peptides, kidney damage, high-fat diet, Nrf2, NLRP3 inflammasome

## Abstract

**Background:**

Oxidative stress and inflammation play important roles in high-fat diet (HFD) induced kidney damage. Previous studies show that the collagen extracted from the skin of monkfish (*Lophius litulon*) with pepsin (pepsin-solubilized collagen, PSC) exhibits good biological activities. This study investigates the protective effect of PSCP against chronic kidney injury in HFD-fed mice.

**Methods:**

Pepsin-solubilized collagen was further hydrolyzed into collagen peptides, and the compound with the best 2,2-diphenyl-1-picrylhydrazyl (DPPH) clearance rate was named pepsin-solubilized collagen peptide (PSCP). A group of mice were fed an HFD for 4 weeks, and then for another 6 weeks PSCP was added to their diet at the amount of either 100 or 200 mg/kg.

**Results:**

Pepsin-solubilized collagen peptide treatment (200 mg/kg) reduced the mice's serum levels of uric acid (UA), creatinine (CRE), and blood urea nitrogen (BUN) by 27, 20, and 37%, respectively. This treatment also remarkably improved renal histopathology. Moreover, the activities of superoxide dismutase (SOD), glutathione peroxidase (GSH-Px), and catalase (CAT) were increased by 96, 52, and 74%, respectively, and decreased the malondialdehyde (MDA) level by 36%. Additionally, PSCP activated the Nrf2 pathway and inhibited NLRP3 signaling to significantly reduce the levels of inflammatory cytokines IL-1β, IL-6, and TNF-α.

**Conclusions:**

Our results indicate that compound PSCP has the potential to prevent or control chronic kidney damage.

## Introduction

With an improvement in living standards, unhealthy lifestyle choices such as a high-fat diet (HFD) and the lack of exercise have significantly increased the incidence of obesity-related metabolic diseases (1). Renal diseases which ultimately lead to renal failure can currently only be treated with a kidney transplant (2). Growing evidence shows a close relationship between obesity and renal dysfunction (3, 4). Moorhead et al. first put forward the concept of lipid nephrotoxicity in 1982 (5). A previous study has shown that a stress signal network composed of ectopic lipid deposition, lipid metabolism disorders, and oxidative stress in the kidney that is linked to obesity conjointly leads to the occurrence and development of chronic kidney disease (CKD) (6). Notably, lipid metabolism disorders, which lead to lipid deposition in the kidney, are one of the common features of many primary and secondary renal diseases (7, 8). In advanced cases, it progresses to CKD. Therefore, lipid metabolism disorders causing CKD have become one of the prime focuses of research studies worldwide. Unfortunately, the involved mechanism is still unclear limiting the treatment strategies.

Oxidative stress and inflammatory response are interrelated in the pathogenesis of kidney disease. An excess of reactive oxygen species (ROS) disrupts the oxidation-antioxidant system causing oxidative stress (9). The Nrf2/HO-1(Nuclear factor erythroid 2-related factor 2/Heme oxygenase-1) pathway is involved in the regulation of oxidative stress responses triggered by internal and external environmental changes (10). Nuclear factor erythroid 2-related factor 2 regulates the activities of antioxidant enzymes and facilitates the elimination of oxygen free radicals by upregulating the downstream HO-1 (11). Studies have shown that ROS is a pro-inflammatory signaling molecule and excessive ROS can lead to chronic inflammation (12, 13). Interestingly, chronic inflammation is another major cause of kidney damage (14). Inflammasomes, a part of the innate immune system, play a central role in the inflammatory response. These can be activated by various stimuli, including adenosine triphosphate, potassium efflux, heme, urate, and ROS, causing secretion of pro-inflammatory effector cytokines and triggering the inflammatory response (15). The inflammasome, first discovered in 2002, is a type of pattern-cognition receptors (16). Nucleotide-binding and oligomerization domain-like receptors protein 3 (NLRP3) may be the best understood inflammasome among the already identified NLRPs. It consists of NLRP3 protein, adapter protein apotosis-associated speck-like protein (ASC), and procaspase-1. After NLRP3 activation, Caspase-1 is activated after the activation of NLRP3, inducing the production and secretion of inflammatory factors such as interleukine-1β (IL-1β) which further aggravates inflammation (17). Overactivation of the NLRP3 inflammasome leads to renal fibrosis, which is one of the causes etiologies of CKD, acute kidney damage, and diabetic nephropathy (18, 19).

Many countries emphasize early recognition and prevention of CKD, mainly to slow the disease progression and reduce complications; however, no particularly effective treatment strategy has yet to be identified (20). Evidence shows that weight loss measures in obese patients can prevent further decline in renal function, but the change is only temporary (21). Therefore, new effective treatments need to be developed. Marine peptide drugs offer obvious advantages against kidney diseases given their small molecular weight, simple structure, fewer side effects, no immunogenicity, and high activity. Therefore, extracting active substances from marine organisms can aid in kidney protection (22–24). Bioactive peptides, are known for their antilipemic properties (25), and those extracted from fish could be an important source for treatments alongside those from milk and eggs (26). The Lophiiformes order (also known as monkfish, anglerfish, and goosefish) is made up of a diverse group of deep-sea fish. They are mostly distributed throughout in the Atlantic Ocean and the Northwestern Pacific Ocean; however, the research on monkfish has been largely focused on those located in the Atlantic (27). Little is known about the population size of monkfish, but the numbers of newly found monkfish species keep growing in the East China Sea (28). Every year approximately 12,000 tons of monkfish is harvested from the East China Sea. The meat is usually made for human consumption, and the by-product skin is often treated as waste or used for making fish food (29). In our previous study, on which this study is based, we evaluated the physicochemical properties of extracts collected from the skin of monkfish (*Lophius litulon*) with pepsin (pepsin-solubilized collagen, PSC) and showed that PSC has good antioxidant and wound healing activity (30). In this study, PSC was hydrolyzed to obtain collagen peptides (pepsin-solubilized collagen peptide, PSCP) with higher antioxidant activity. Furthermore, we evaluated the renal protective effect of PSCP on HFD-fed mice and found that PSCP functions by activating the Nrf2 pathway and inhibiting the NLRP3 inflammatory signaling. Our results suggest that PSCP may be an effective treatment for CKD.

## Materials and Methods

### Preparation of PSCP From the Skin of Monkfish

Previously, we used PSC from the skin of monkfish and studied its physical and chemical properties (30). Based on that research, we prepared PSC through acid protease hydrolysis under optimal conditions (50°C, pH 3.0, for 5 h) to determine the compound with the best DPPH free radical scavenging rate, which was then named PSCP. We selected acid protease to hydrolyze monkfish skin based on its efficiency. Unlike in our previously published research, we found neutrase to be the best enzyme for preparing peptides derived from monkfish meat (31). This may be attributed to the disparities in the protein composition.

### Animals and Treatments

Thirty-two 4-weeks-old male C57BL/6J mice were provided by the Experimental Animal Center of Zhejiang Province (Hangzhou, China). The animal protocol, animal certificate No. SCXK (ZHE 2014-0001), was approved by the Experimental Animal Ethics Committee of Zhejiang Ocean University (Zhoushan, China). Animals were kept under controlled environmental conditions of 22 ± 2°C, 55 ± 5% humidity, and a normal light/dark (12 h/12 h) cycle. After 7 days of adaptive feeding, eight mice were fed a normal diet (control), while the other 24 mice were fed a HFD based on lard (containing 60% kcal from fat, 4,057 kcal/kg; Research Diets, Inc. USA) for 4 weeks in order to foster their development of obesity. Next, the animals on an HFD were randomly divided into three groups with eight mice per group and were fed three distinct diets (PSCP in all groups administered through gavage) for the following 6 weeks' period (PSCP in all groups was administered through gavage). The diets were as follows: (1) unchanged HFD (HFD group); (2) HFD with 100 mg/kg PSCP (100 PSCP group); and (3) HFD with 200 mg/kg PSCP (200 PSCP group). The weights of the mice were recorded every other day. At the end of the tenth week, the mice were euthanized after 12 h of fasting, and serum and tissue samples were harvested and stored at −80°C. The organ index was calculated as the organ weight divided by the mouse weight × 100%.

### Biochemical Analysis

To obtain serum, the blood samples were centrifuged (10,000 rpm) at 4°C for 5 min. One gram of kidney tissue was mixed on ice with 9 ml of normal saline and then centrifuged (4,000 rpm) for 10 min. The protein concentration of the kidney homogenate supernatant was quantified using a BCA kit (Solarbio, Beijing, China). The serum levels in the kidney of uric acid (UA), creatinine (CRE), and blood urea nitrogen (BUN) as well as malondialdehyde (MDA), superoxide dismutase (SOD), glutathione peroxidase (GSH-Px), catalase (CAT), triglycerides (TG), and total cholesterol (TC) were determined based on instructions from Nanjing Jiancheng Bioengineering Institute (Nanjing, China).

### Measurement of Inflammatory Cytokines in the Kidney

A 10% kidney homogenate was prepared in PBS (instead of normal saline) as described in Section Biochemical Analysis. The levels of IL-1β, interleukin-6 (IL-6), and tumor necrosis factor-α (TNF-α) were quantified using the corresponding ELISA kits (Elabscience Biotechnology, Inc., Wuhan, China) following the manufacturer's instructions.

### Histopathological Analysis and Immunohistochemistry Assay

The mouse kidneys were fixed in 4% neutral formalin for 48 h. The fixed kidney and liver tissues were embedded with paraffin according to the standard procedure for dehydration, transparency, and wax immersion. A 5 μm thick tissue section was prepared for Hematoxylin-eosin (H&E), Periodic Acid-Schiff (PAS), Masson, and immunohistochemistry (IHC) staining following the standard protocols. Photomicrographs were observed under an optical microscope (Biological microscope CX31, Olympus, Japan) and photographed at 400× magnification. We used the antibody for Collagen Type I (1:1,000, Rabbit, Proteintech).

### Western Blotting

Kidney tissue was snap-frozen in liquid nitrogen and crushed into powder. The collected powder was subjected to Western blotting as described by Zheng et al. (32). Each kidney tissue sample contained 50 μg of protein. The detection was performed on the FluorChem FC3 system (ProteinSimple, Waltham, MA, USA) using the enhanced chemiluminescence reagent (ECL, Solarbio, Beijing, China). Image Lab software was used to analyze protein level, and β-actin was used as an internal control. The following antibodies were used: NLRP3 (1:1,000, Rabbit, Cell Signaling Technology), HO-1 (1:2,000, Mouse, Proteintech), GCLM (1:2,000, Rabbit, Proteintech), Nrf2 (1:1,000, Rabbit, Proteintech), NQO1 (1:5,000, Mouse, Proteintech), Caspase-1 (1:1,000, Rabbit, Proteintech), cleaved-Caspase-1 (1:1,000, Rabbit, Affinity), ASC (1:1,000, Rabbit, Cell Signaling Technology), β-actin (1:5,000, Mouse, Affinity).

### Statistical Analysis

Each experiment was repeated at least three times. Data were analyzed by analysis of variance (ANOVA) using the SPSS 22.0 software (IBM^®^ SPSS^®^ Statistics, Ehningen, Germany) and are expressed as the mean ± SD (*n* = 8). The *P*-value < 0.05 denotes significant difference.

## Results

### Effect of PSCP on the Body Weight and Organ Index in HFD-Fed Mice

**Figure 1:**
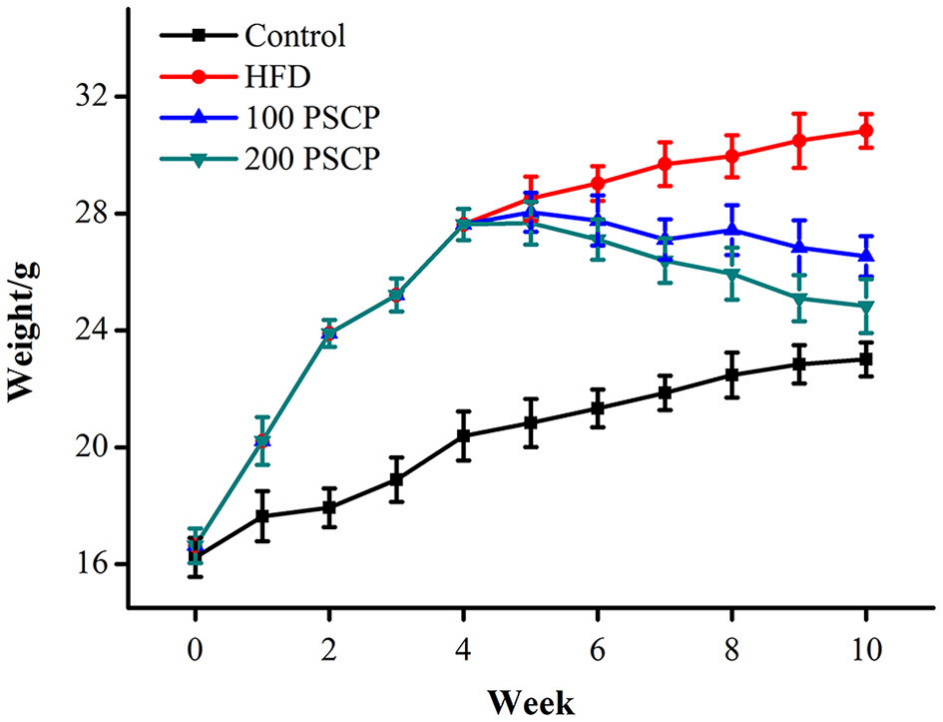
Effect of PSCP on body weight in HFD-fed mice. Data are presented as mean ± SD (*n* = 8).

Mice body weights were measured every other day, and the weekly weight change was used as an indicator for the effect of PSCP in HFD-fed mice. As shown in Figure 1, after 4 weeks of HFD feeding, the average bodyweight of mice in the HFD group was significantly higher than that of the mice in the control. However, after treatment with PSCP at Week 5, mice started to lose weight. In general, changes in organ weight is considered to be a more sensitive parameter than histopathological changes. The kidney index of the mice in the HFD group was significantly higher than that of the mice in the control group (*p* < 0.01). After PSCP treatment, the kidney index of the HFD-fed mice (*p* < 0.05, Table 1) was significantly lowered, indicating PSCP's effectiveness in reducing HFD induced kidney damage. Compared with the control group, the liver index of the mice in the HFD group increased by 35%. After treatment with 100 mg/kg PSCP and 200 mg/kg PSCP, the liver index decreased by 14 and 30%, respectively.

**Table 1:** Effect of PSCP on liver and kidney index of mice.

	**Control**	**HFD**	**100 mg/kg PSCP**	**200 mg/kg PSCP**
Liver index (%)	4.66 ± 0.22^##^	6.38 ± 0.09^**^	5.58 ± 0.02^*^^#^	4.92 ± 0.02^*^^#^
Kidney index (%)	1.31 ± 0.07^##^	1.47 ± 0.08^**^	1.39 ± 0.05	1.36 ± 0.04^#^

*Data are given as mean ± SD (n = 8)*.

* *p < 0.05*,

** *p < 0.01 vs. Control group*.

# *p < 0.05*,

## *p < 0.01 vs. HFD group*.

### Effect of PSCP on the Levels of UA, CRE, and BUN in HFD-Fed Mice

Uric acid, CRE, and BUN levels are important clinical biochemical indicators to determine renal dysfunction (33, 34). We estimated these indicators to evaluate the protection degree of PSCP against HFD induced kidney injury. As shown in Table 2, compared with the control group, the serum levels of UA, CRE, and BUN were significantly higher in the HFD group (*p* < 0.01), indicating HFD induced renal dysfunction. However, these levels were significantly reduced (*p* < 0.05) in this same group after undergoing PSCP treatment. This again supports the hypothesis that PSCP can effectively improve HFD-induced kidney damage.

**Table 2:** Effect of PSCP on serum UA (A), CRE (B), and BUN (C) in HFD-fed mice.

	**Control**	**HFD**	**100 mg/kg PSCP**	**200 mg/kg PSCP**
UA (μmol/L)	6.94 ± 1.09^##^	17.22 ± 0.96^**^	14.65 ± 0.63^**^^#^	12.60 ± 0.96^**^^##^
CRE (μmol/L)	13.12 ± 0.88^##^	19.379 ± 1.05^**^	17.286 ± 0.91^**^^#^	15.580 ± 0.57^*^^##^
BUN (mmol/L)	9.36 ± 0.61^##^	18.87 ± 0.72^**^	14.92 ± 0.76^**^^##^	11.80 ± 0.75^**^^##^

*Data are given as mean ± SD (n = 8)*.

* *p < 0.05*,

** *p < 0.01 vs. the Control group*.

# *p < 0.05*,

## *p < 0.01 vs. HFD group*.

### Effect of PSCP on Kidney Injury in HFD-Fed Mice

**Figure 2:**
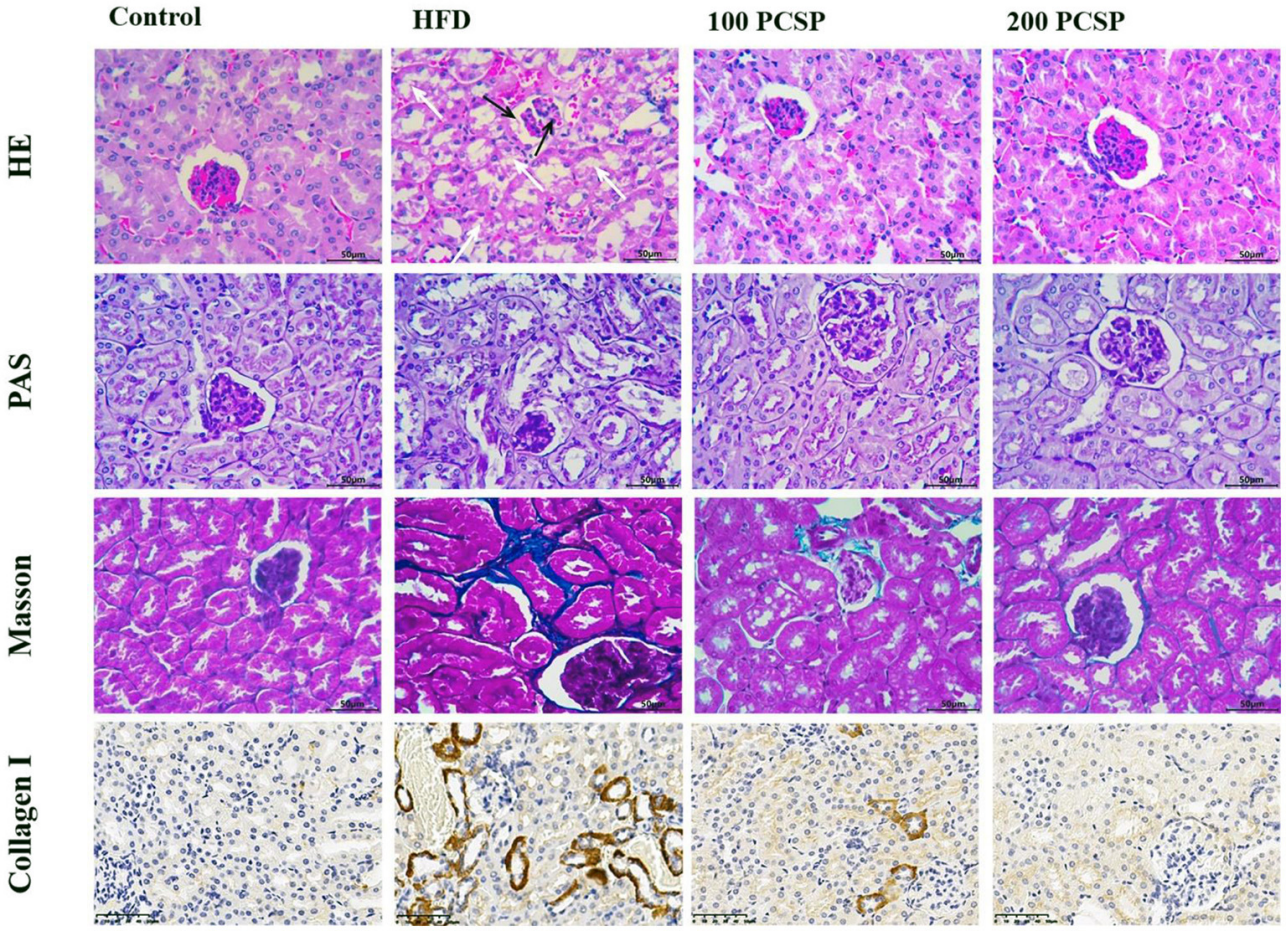
Effect of PSCP on morphological characteristics in the kidneys of HFD-fed mice (magnification ×400). HE staining, PAS staining, Masson staining, and collagen II HC staining were performed to detect morphology, glycogen, and collagen, respectively. The white arrow represents tubular damage and the black arrow represents glomerular damage.

To further investigate the protective effect on PSCP, we performed a histopathological analysis using H&E, PAS, Masson, and IHC staining of the mice's kidney tissue (Figure 2). The H&E staining of kidney tissue showed normal renal morphology in the mice of the control group. The renal corpuscles, composed of renal sacs surrounded by glomeruli, presented clear lumen, regular renal tubular morphology, and showed no inflammatory cell infiltration. On the contrary, the HFD group showed glomeruli atrophy, enlargement of the cyst cavity, proliferation of mesangial cells and mesangial matrix, unclear renal tubule structure with obvious vacuolar degeneration, and noticeable inflammatory cell infiltration. However, treatment of this group with 100 mg/kg PSCP inhibited the proliferation of mesangial cells and mesangial matrix as well as alleviated the vacuolar degeneration of renal tubules. Furthermore, treatment with 200 mg/kg PSCP helped the renal corpuscles revert to healthy (normal) glomerulus and renal tubules morphology without any sign of inflammatory cell infiltration.

In the PAS staining (Figure 2), the kidney tissue structure of the mice in the control group was normal, showing a small mesangial area, normal glomerular volume, and no mesangial hyperplasia. In the HFD group, the kidney tissue staining (positive) was significantly deepened, showing highly proliferated mesangium and an increased mesangial area. However, with an increased dose of PSCP, the glomerular surface area and mesangial area decreased significantly in the PSCP-treated HFD-fed mice.

Masson staining can detect collagen deposition in kidney tissue. Through this staining method, we found that kidney tissue of the mice in the HFD group showed renal tubular interstitial fibrosis. Immunohistochemistry results of type I collagen showed a large amount of collagen deposition around renal tubules compared to the normal group. However, after the administration of PSCP, the kidney damage in the HFD-fed mice was ameliorated. Overall, the above-mentioned histopathological analyses suggest that PSCP therapy can improve and reverse the renal structural changes to reduce kidney damage in HFD-fed mice.

### Effect of PSCP on Liver Injury in HFD-Fed Mice

**Figure 3:**
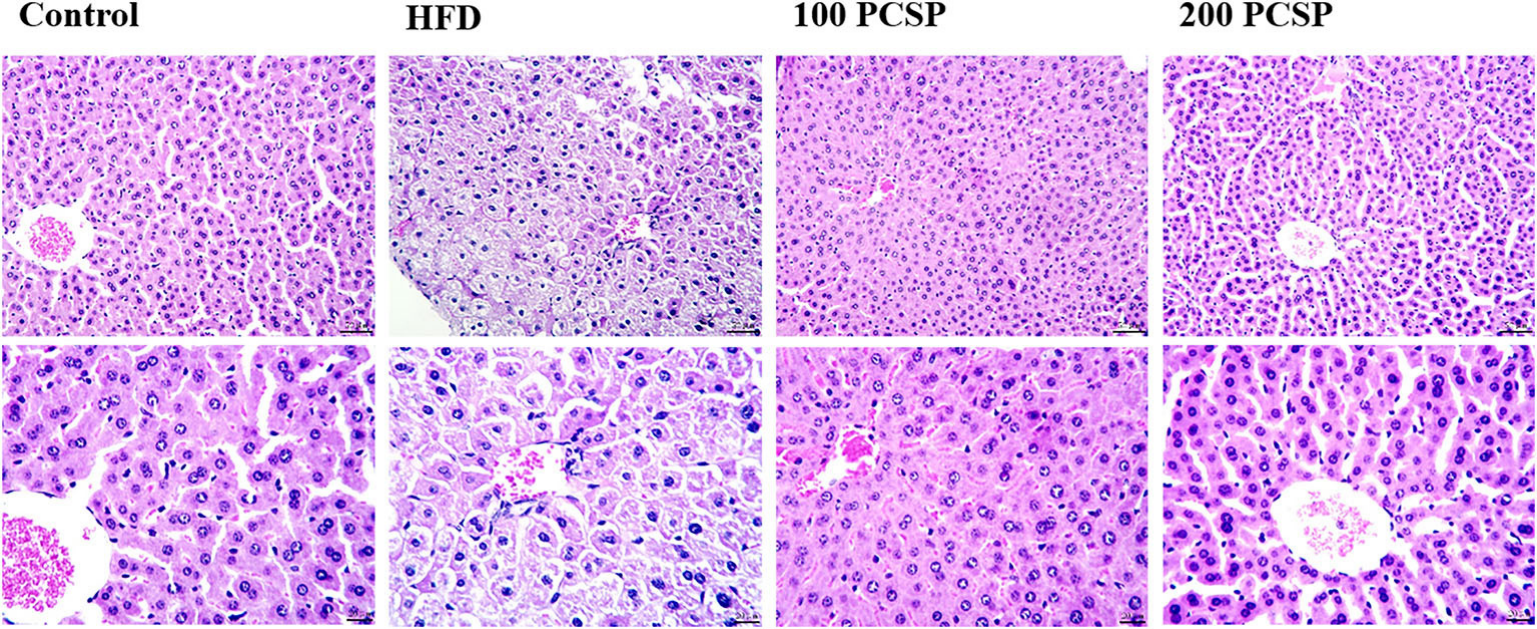
Effect of PSCP on morphological characteristics in the livers of HFD-fed mice (magnification ×200 and ×400). Representative photomicrograph of HE staining.

H&E staining of liver tissue (Figure 3) showed clear lobule structure, regular morphology of liver cells and orderly arrangement of liver cords in control mice. In the HFD group, hepatic cords were confused, hepatic cell margins were blurred, steatosis was observed, there were a lot of vacuoles in the liver cells, and inflammatory cells were infiltrated near the central vein. Compared with the HFD group, the hepatic lobules treated with 100 mg/kg PSCP had clear structure, regular hepatic cell morphology, and orderly hepatic cord arrangement. Treatment with 200 mg/kg PSCP the hepatic lobule structure was clear, the hepatic cord was arranged neatly, and the hepatic cell morphology was regular, almost close to the normal group.

### Effect of PSCP on Blood Lipids in HFD-Fed Mice

To investigate the effect of PSCP on blood lipids, we examined serum TC and TG levels. We found that the TC content in the serum of HFD-fed mice was increased by 103% (*p* < 0.05, Table 3), and TG was elevated by 211%. The results showed that the serum lipid level of HFD-fed mice increased significantly. Notably, PSCP treatment improved TG and TC, indicating that PSCP can substantially reduce blood lipid in HFD-fed mice.

**Table 3:** Effect of PSCP on serum TC and TG in HFD-fed mice.

	**Control**	**HFD**	**100 mg/kg PSCP**	**200 mg/kg PSCP**
TC (mmol/L)	3.57 ± 1.55^##^	7.24 ± 1.43^**^	4.44 ± 0.38^##^	3.66 ± 0.31^##^
TG (mmol/L)	1.29 ± 0.39^##^	4.01 ± 0.10^**^	2.09 ± 0.45^*^^##^	1.66 ± 0.38^##^

*Data are given as mean ± SD (n = 8)*.

* *p < 0.05*,

** *p < 0.01 vs. Control group*,^#^*p < 0.05*,

## *p < 0.01 vs. HFD group*.

### Effect of PSCP on MDA and Antioxidant Enzyme in HFD-Fed Mice

To examine the effect of PSCP on oxidative stress, we estimated the levels of MDA and antioxidant enzymes in the kidney. We found that the MDA content in the kidney of HFD-fed mice was elevated by 118% (*p* < 0.01, Table 4), while the activities of SOD, GSH-Px, and CAT were decreased by 56, 38, and 48% (*p* < 0.01), respectively. This indicates an unbalanced antioxidant system in the kidney and a state of HFD-induced oxidative stress. Interestingly, PSCP treatment reverted the levels of MDA (*p* < 0.01), SOD, GSH-Px, and CAT (*p* < 0.05) back to their normal levels. This shows that PSCP was significantly effective in reducing the oxidative stress in the kidneys of HFD-fed mice.

**Table 4:** Effect of PSCP on the levels of renal MDA (A), SOD (B), GSH-Px (C), and CAT (D) in HFD-fed mice.

	**Control**	**HFD**	**100 mg/kg PSCP**	**200 mg/kg PSCP**
MDA (nmol/mgprot)	7.61 ± 1.05^##^	16.59 ± 1.1.1^**^	12.38 ± 0.82^**^^##^	10.59 ± 0.78^*^^##^
SOD (U/mgprot)	154.85 ± 9.96^##^	67.85 ± 10.43^**^	96.91 ± 9.49^**^^#^	132.78 ± 8.86^##^
GSH-Px (U/mgprot)	226.15 ± 12.31^##^	139.18 ± 10.43^**^	160.38 ± 11.54^**^	210.87 ± 5.28^##^
CAT (U/mgprot)	14.42 ± 1.33^##^	7.53 ± 1.25^**^	11.63 ± 1.35^*^^##^	13.09 ± 0.66^##^

*Data are given as mean ± SD (n = 8)*.

* *p < 0.05*,

** *p < 0.01 vs. Control group*,

# *p < 0.05*,

## *p < 0.01 vs. HFD group*.

### Effect of PSCP on Inflammatory Cytokines in HFD-Fed Mice

Interleukin-6, IL-1β, and TNF-α are the common cellular inflammatory cytokines used for clinical investigation. Studies have shown that glomerular mesangial cells aggressively produce inflammatory mediators such as IL-6 and IL-1β during impaired renal function. This aggravates glomerular sclerosis causing a decline of renal function (35–37). To determine the effect of PSCP on inflammatory cytokines, we estimated the renal levels of IL-6, IL-1β, and TNF-α. As shown in Table 5, kidney IL-1β, IL-6, and TNF-α increased in the HFD group compared with the control group (*p* < 0.01) by 173, 197, and 111%, respectively. However, PSCP treatment (200 mg/kg) reduced the level of IL-1 β, IL-6, and TNF-α by 64, 58, and 49%, respectively, compared with the model group, alleviating renal inflammation in HFD-fed mice.

**Table 5:** Effect of PSCP on the levels of renal IL-1β (A), IL-6 (B), and TNF-α (C) in HFD-fed mice.

	**Control**	**HFD**	**100 mg/kg PSCP**	**200 mg/kg PSCP**
IL-1β (pg/ml)	33.58 ± 2.29^##^	91.65 ± 3.05^**^	49.59 ± 3.23^**^^##^	33.20 ± 2.27^##^
IL-6 (pg/ml)	23.04 ± 1.99^##^	68.45 ± 2.75^**^	41.16 ± 2.22^**^^##^	28.91 ± 1.73^*^^##^
TNF-α (pg/ml)	52.29 ± 3.24^##^	110.09 ± 3.79^^*^^*^^	76.96 ± 3.34^^*^^*^^^##^	56.19 ± 3.84^##^

*Data are given as mean ± SD (n = 8)*.

* *p < 0.05*,

** *p < 0.01 vs. Control group*, ^#^*p < 0.05*,

## *p < 0.01 vs. HFD group*.

### Effect of PSCP on Nrf2-Mediated Antioxidant Response in HFD-Fed Mice

**Figure 4:**
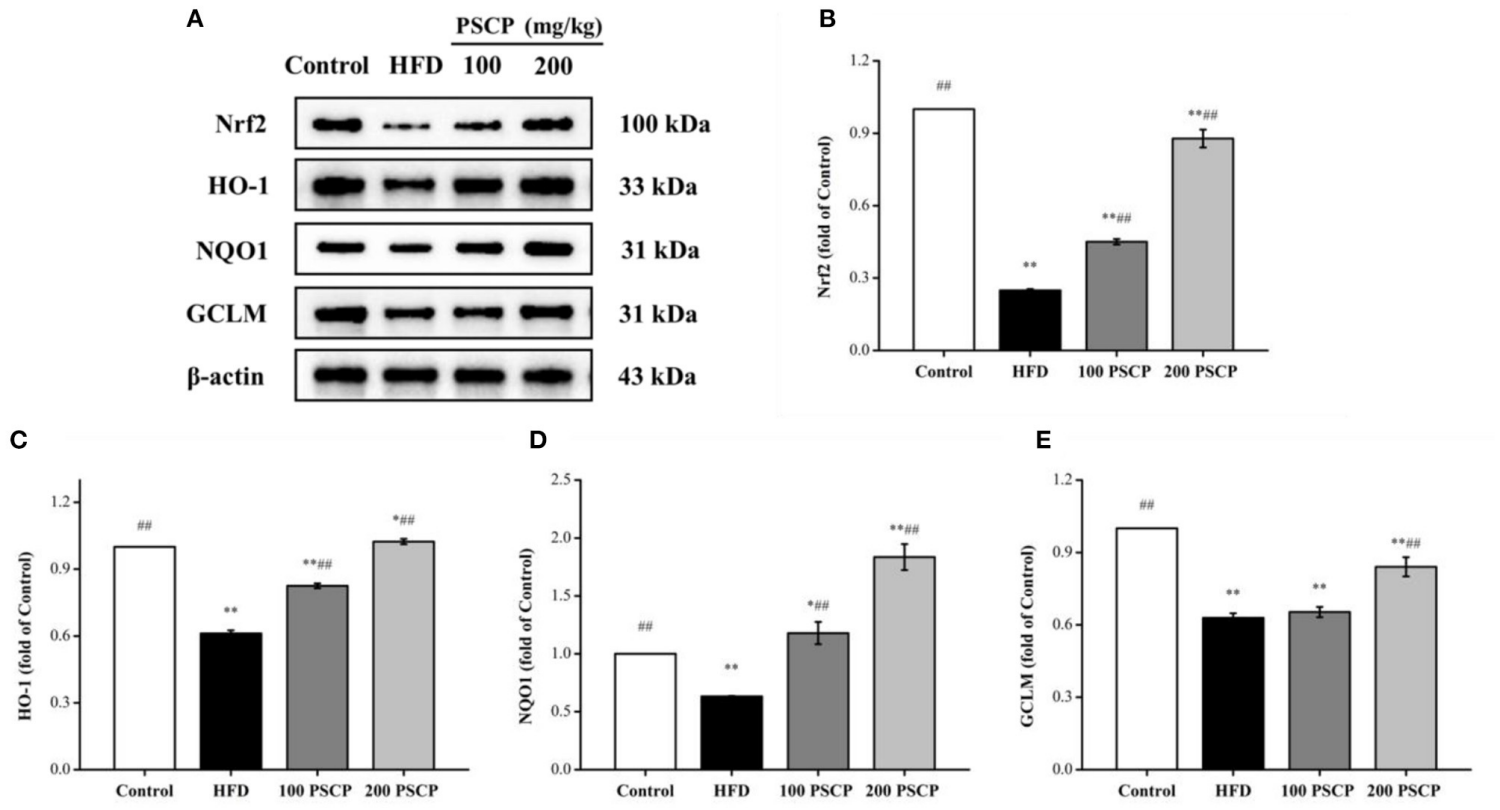
Effect of PSCP on Nrf2-mediated antioxidant response in HFD-fed mice. **(A)** Western blot analysis of Nrf2, HO-1, NQO1, and GCLM in kidney; **(B)** Quantitative analysis of Nrf2; **(C)** Quantitative analysis of HO-1; **(D)** Quantitative analysis of NQO1; **(E)** Quantitative analysis of GCLM. ^*^p < 0.05, ^**^p < 0.01 vs. Control group, ^#^*p* < 0.05, ^##^*p* < 0.01 vs. HFD group.

To further examine the potential anti-oxidation mechanism of PSCP in HFD-induced kidney injury, we used Western blotting to estimate the protein levels of Nrf2 and HO-1, nicotinamide quinone oxidoreductase 1 (NQO1), and glutamate-cysteine ligase regulatory subunit (GCLM). Notably, the Nrf2 signaling pathway can regulate cell redox homeostasis by up-regulating several antioxidant proteins, which ease cellular oxidative stress (38). As shown in Figure 4, compared to the control group, the protein levels of Nrf2, HO-1, NQO1, and GCLM were decreased in the HFD group (*p* < 0.01) by 75, 38, 37, and 37%, respectively. However, these protein levels increased in the group that received PSCP treatment (200 mg/kg) in comparison to the HFD group (*p* < 0.05) by 253, 68, 190, and 34%, respectively. This suggests that PSCP potentially functions via the activation of the Nrf2/HO-1 signaling pathway.

### Effect of PSCP on NLRP3 Inflammatory Pathway in HFD-Fed Mice

**Figure 5:**
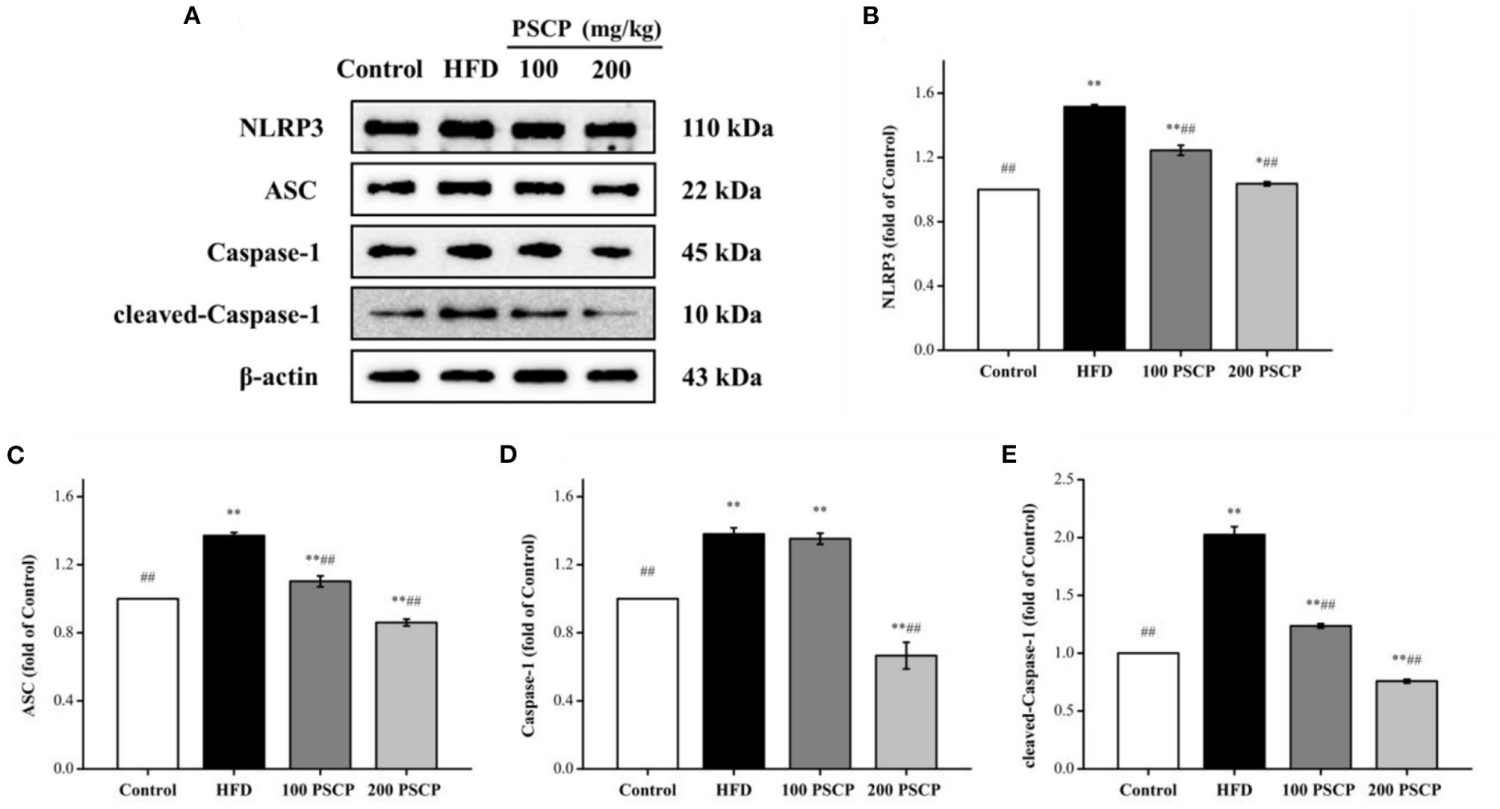
Effect of PSCP on NLRP3 inflammatory pathway in HFD-fed mice. **(A)** Western blot analysis of NLRP3, ASC, Caspase-1, and cleaved-Caspase-1 in kidney; **(B)** Quantitative analysis of NLRP3; **(C)** Quantitative analysis of ASC; **(D)** Quantitative analysis of Caspase-1; **(E)** Quantitative analysis of cleaved-Caspase-1. ^*^p < 0.05, ^**^p < 0.01 vs. Control group, ^#^*p* < 0.05, ^##^*p* < 0.01 vs. HFD group.

The NLRP3 signaling pathway is a typical inflammatory signaling pathway that mainly involves the NLRP3 inflammasome, an apoptosis-associated speck-like protein containing a CARD (ASC), and pro-Caspase-1 (39, 40). To further understand the mechanism of the PSCP protective effect on HFD-induced kidney injury, we focused on the NLRP3 inflammation signaling pathway. As shown in Figure 5, the protein levels of NLRP3 inflammasome-related proteins (NLRP3, ASC, Caspase-1, cleaved-Caspase-1) were increased in the kidney of the HFD group compared to in the control group (*p* < 0.01) by 52, 37, 38, and 100%, respectively. However, these protein levels were reduced after undergoing PSCP treatment (200 mg/kg) (*p* < 0.05) by 32, 37, 52, and 63%, respectively. This indicates that PSCP can prevent kidney inflammation by inhibiting the activation of the NLRP3 inflammasome.

## Discussion

In this study, long-term HFD feeding was used to establish a mice disease model for CKD. The HFD caused significant vacuolar degeneration of renal tubules and led to elevated serum levels of CRE and BUN, indicating renal dysfunction in HFD-fed mice. However, the addition of PSCP to the mice's diet showed a protective effect against CKD. Pepsin-solubilized collagen peptide treatment significantly reduced the elevated serum levels of UA, CRE, and BUN in HFD-fed mice. Immunohistochemical examination also indicated improved kidney histopathology. Overall, both biochemical and histopathological evaluations indicated that PSCP can ameliorate HFD-induced CKD in mice.

Our results have shown that HFD leads to a large amount of collagen deposition around the renal tubules, which could be explained as renal fibrosis. This massive deposition of collagen can be alleviated by feeding PSCP, indicating PSCP's protective effects on renal fibrosis. Long-term HFDs are known to elevate renal ROS, an important endothelial damage factor (41). Studies have shown that hyperlipidemia leads to increased ROS production in monocytes. These studies indicate that oxidative stress could be the prime cause of renal dysfunction. Therefore, the protective effect of the renal antioxidant defense system is critical (42). Notably, the activities of SOD, GSH-Px, and CAT are indicators of the cellular antioxidant capacity. Meanwhile, MDA, which is produced by lipid peroxidation, reflects the degree of free radical damage (43). We found that PSCP treatment effectively reduced the MDA content in the kidney of HFD-fed mice and restored the activities of SOD, GSH-Px, and CAT. Nuclear factor erythroid 2-related factor 2, an important redox-sensitive transcription factor, is involved in the cellular redox balance (44), and the Nrf2 signaling pathway is closely related to kidney diseases including acute kidney damage (45), CKD (46), and diabetic nephropathy (47). Nezu et al. suggests that the activation of Nrf2 has a preventive effect on kidney disease, therefore Nrf2 activators can be useful for the treatment of kidney diseases (48). Our study shows that PSCP treatment can improve the protein levels of Nrf2, HO-1, NQO1, and GCLM in HFD-induced kidney damage. These results suggest that PSCP functions by countering cellular oxidative stress.

Inflammation is the main factor in most acute and CKDs. The release of IL-1β induces the chemotaxis of neutrophils into the inflammation site. Other inflammatory factors such as TNF-α and IL-6 then promote the proliferation of the glomerular mesangial matrix (49). Through this study, we show that PSCP treatment significantly decreases the levels of TNF-α, IL-6, and IL-1β. This suggests that PSCP functions via inhibiting the secretion of inflammatory factors to reduce the inflammatory response in the kidney of HFD-fed mice. During lipid nephrotoxicity, inflammation leads to mitochondrial dysfunction causing cell damage. The NLRP3 inflammasome, a multi-protein complex of NLRP3, ASC, and pro-Caspase-1, serves as a platform for mediating the activation of Caspase-1. Caspase-1-mediated canonical pyroptosis may enhance the release of pro-inflammatory cytokines such as IL-1β and IL-18. Activated NLRP3 inflammasome is involved in the pathogenesis of many kidney diseases, such as acute kidney damage, calcium oxalate crystal nephropathy, and CKD (39). However, the exact mechanism of HFD induced NLRP3 inflammasome activation in renal tubular epithelial cells remains elusive and needs further study (49, 50). Some studies indicate that the NLRP3 inflammasome is activated by ROS in human corneal epithelial cells and human aortic endothelial cells. In this study, we have shown that HFD can activate the NLRP3 inflammasomes in the kidney (51). Interestingly, PSCP treatment significantly downregulated the NLRP3 and related proteins ASC, pro-Caspase-1, and Caspase-1. This suggests that PSCP protects from HFD-induced kidney damage by blocking the secretion of inflammatory factors and inhibiting the activation of NLRP3 inflammasomes. Our future work will be focused on studying the protective mechanism of PSCP in HFD-induced pyroptosis.

## Conclusions

Our results indicate that PSCP has a protective effect against HFD-induced CKD in mice. Pepsin-solubilized collagen peptide functions by preventing HFD-induced oxidative stress. It inhibits ROS-mediated activation of NLRP3 inflammasomes which in turn reduces inflammation. Based on these results, we suggest that PSCP may be an effective therapy for CKD.

## Data Availability Statement

The raw data supporting the conclusions of this article will be made available by the authors, without undue reservation.

## Ethics Statement

The animal study was reviewed and approved by Experimental Animal Ethics Committee of Zhejiang Ocean University.

## Author Contributions

JZ and ZY: conceptualization. BM, GZ, XT, and FY: formal analysis. JZ and BM: investigation and writing—original draft preparation. JZ, BM, and WZ: data curation. FY: writing—review and editing. ZY: funding acquisition. All authors have read and agreed to the published version of the manuscript.

## Funding

This research was funded by Zhejiang Provincial Public Welfare Technology Research Program, grant number LGN21D060002 to ZY and National Science Foundation, grant number 82073764 to FY.

## Conflict of Interest

The authors declare that the research was conducted in the absence of any commercial or financial relationships that could be construed as a potential conflict of interest.

## Publisher's Note

All claims expressed in this article are solely those of the authors and do not necessarily represent those of their affiliated organizations, or those of the publisher, the editors and the reviewers. Any product that may be evaluated in this article, or claim that may be made by its manufacturer, is not guaranteed or endorsed by the publisher.
