# Supplementary figures and images for: Using Collagen Peptides From the Skin of Monkfish (Lophius litulon) to Ameliorate Kidney Damage in High-Fat Diet Fed Mice by Regulating the Nrf2 Pathway and NLRP3 Signaling
Source: Front Nutr. 2022 Feb 10;9:798708. doi: 10.3389/fnut.2022.798708 (PMC8866304; doi:10.3389/fnut.2022.798708)

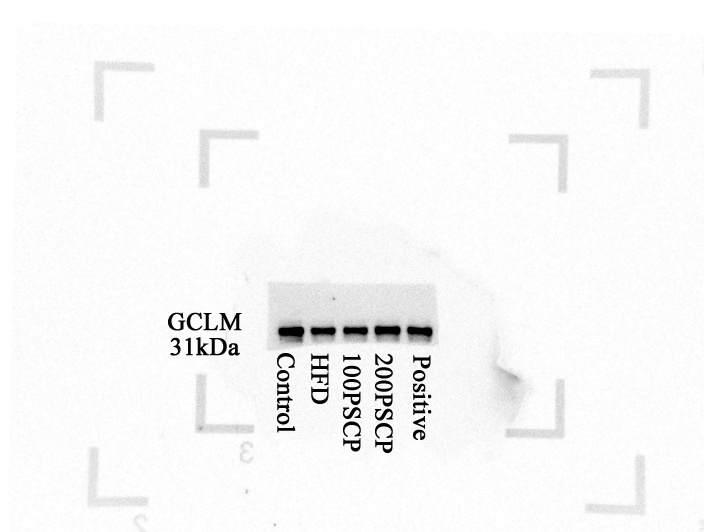

Supplement: Supplementary file 1 [file Data_Sheet_1.ZIP › Original data/Western blots-Fig6/GCLM/GCLM-Repeat 1.tif]

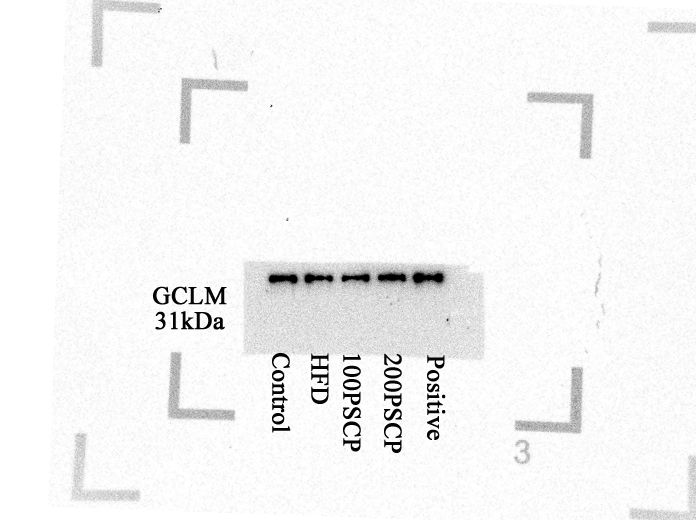

Supplement: Supplementary file 1 [file Data_Sheet_1.ZIP › Original data/Western blots-Fig6/GCLM/GCLM-Repeat 2.tif]

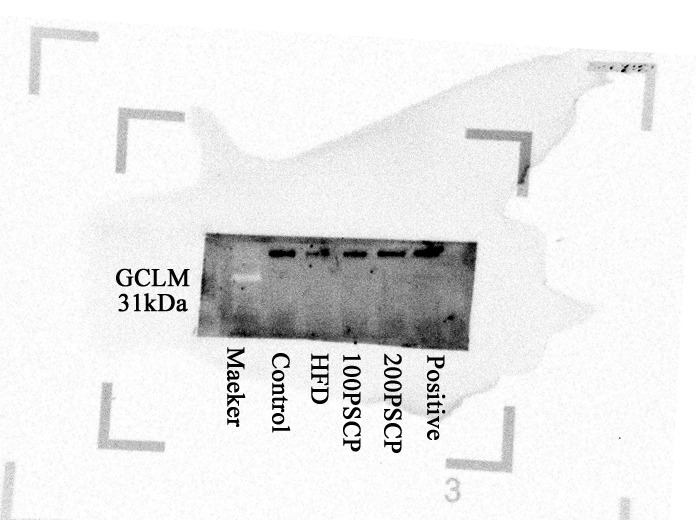

Supplement: Supplementary file 1 [file Data_Sheet_1.ZIP › Original data/Western blots-Fig6/GCLM/GCLM-Repeat 3.tif]

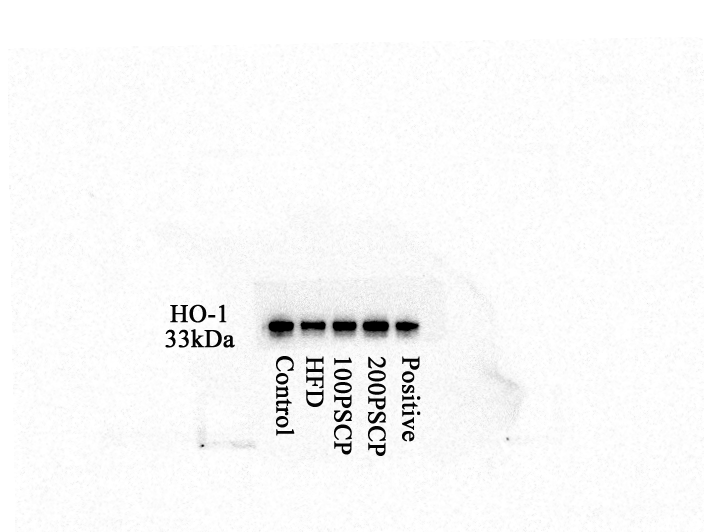

Supplement: Supplementary file 1 [file Data_Sheet_1.ZIP › Original data/Western blots-Fig6/HO-1/HO-1 -Repeat 1.tif]

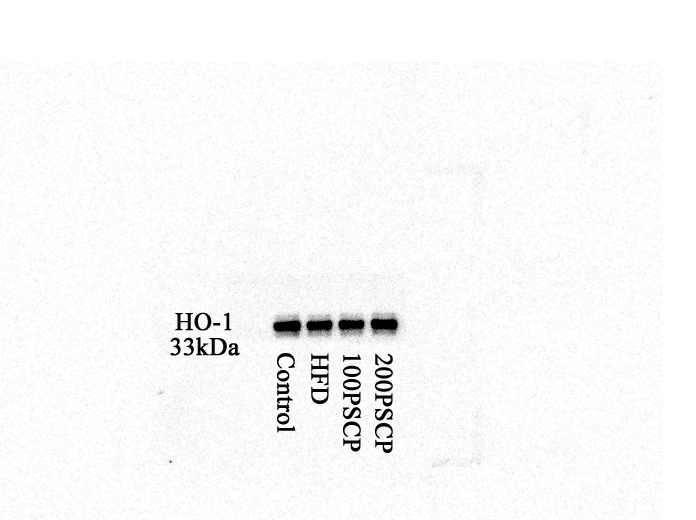

Supplement: Supplementary file 1 [file Data_Sheet_1.ZIP › Original data/Western blots-Fig6/HO-1/HO-1 -Repeat 2.tif]

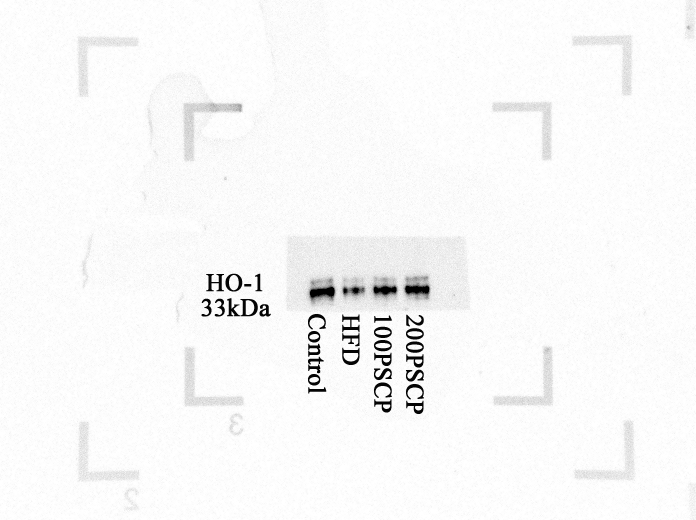

Supplement: Supplementary file 1 [file Data_Sheet_1.ZIP › Original data/Western blots-Fig6/HO-1/HO-1 -Repeat 3.tif]

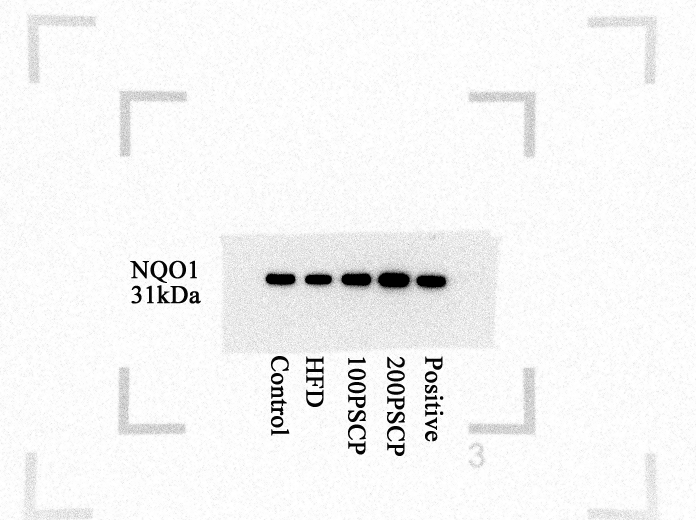

Supplement: Supplementary file 1 [file Data_Sheet_1.ZIP › Original data/Western blots-Fig6/NQO1/NQO1 -Repeat 1.tif]

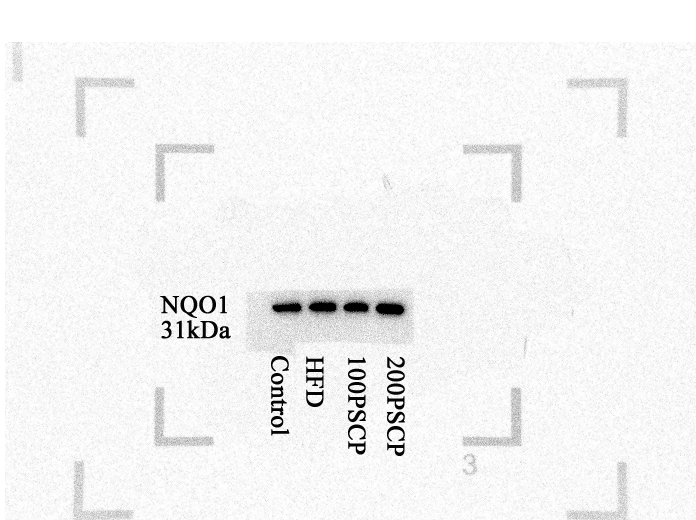

Supplement: Supplementary file 1 [file Data_Sheet_1.ZIP › Original data/Western blots-Fig6/NQO1/NQO1 -Repeat 2.tif]

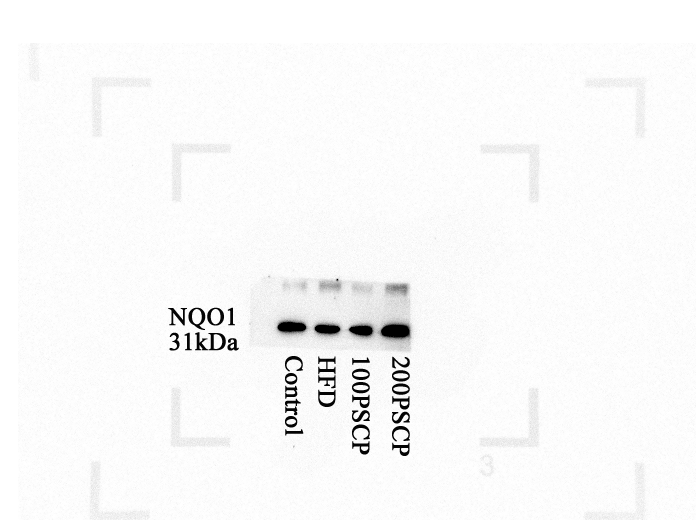

Supplement: Supplementary file 1 [file Data_Sheet_1.ZIP › Original data/Western blots-Fig6/NQO1/NQO1 -Repeat 3.tif]

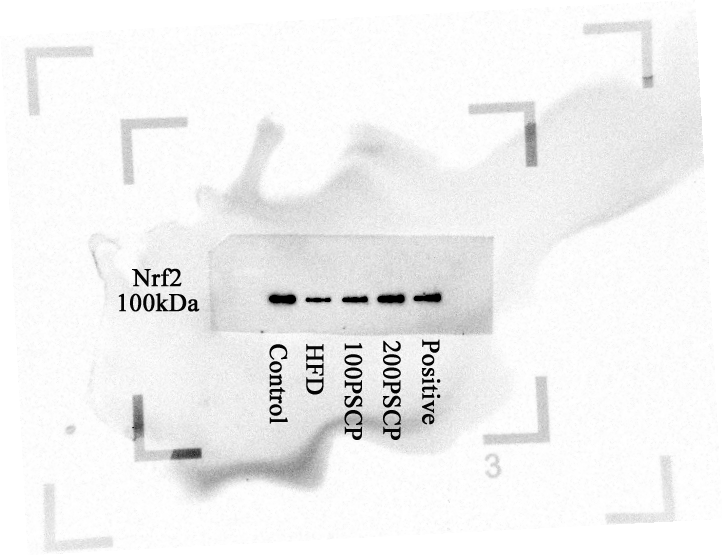

Supplement: Supplementary file 1 [file Data_Sheet_1.ZIP › Original data/Western blots-Fig6/Nrf2/Nrf2-Repeat 1.tif]

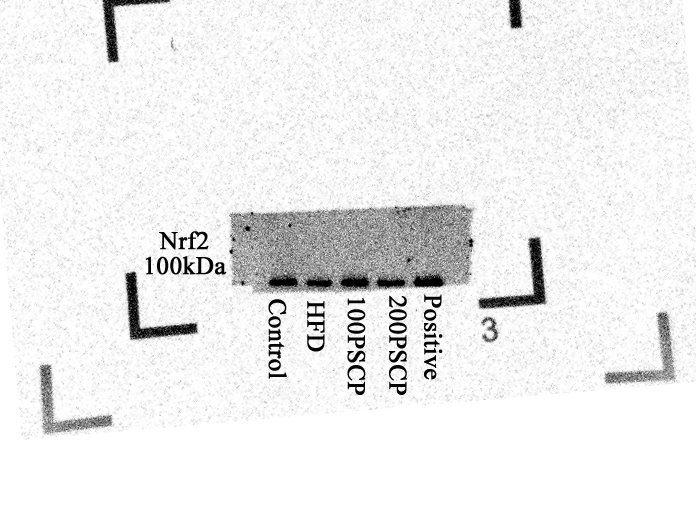

Supplement: Supplementary file 1 [file Data_Sheet_1.ZIP › Original data/Western blots-Fig6/Nrf2/Nrf2-Repeat 2.tif]

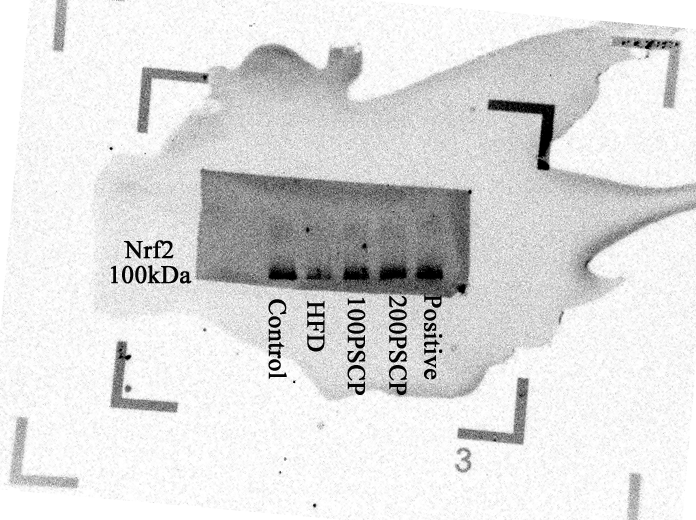

Supplement: Supplementary file 1 [file Data_Sheet_1.ZIP › Original data/Western blots-Fig6/Nrf2/Nrf2-Repeat 3.tif]

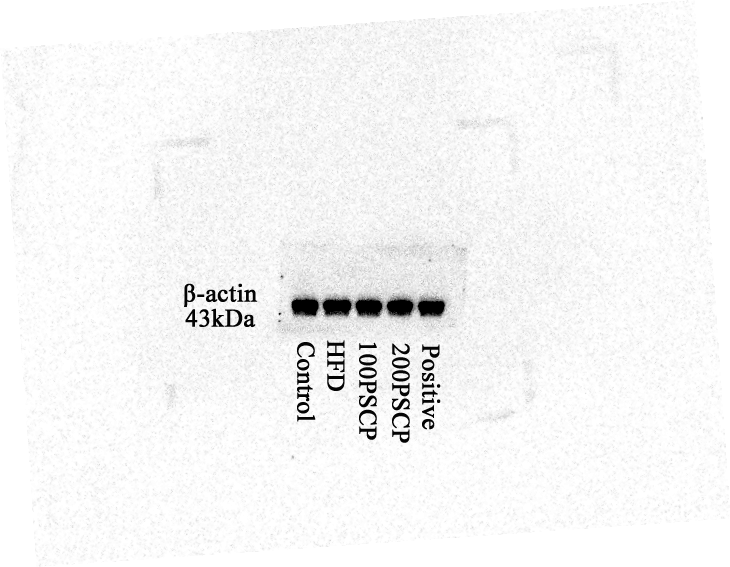

Supplement: Supplementary file 1 [file Data_Sheet_1.ZIP › Original data/Western blots-Fig6/a┬-actin/a┬-actin-Repeat 1.tif]

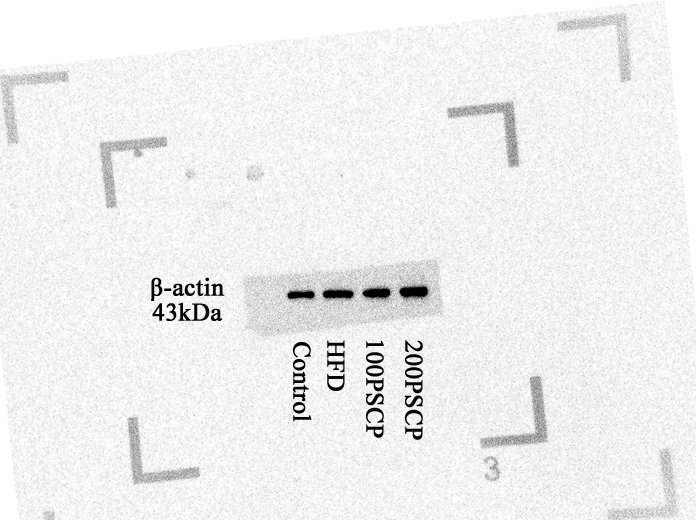

Supplement: Supplementary file 1 [file Data_Sheet_1.ZIP › Original data/Western blots-Fig6/a┬-actin/a┬-actin-Repeat 2.tif]

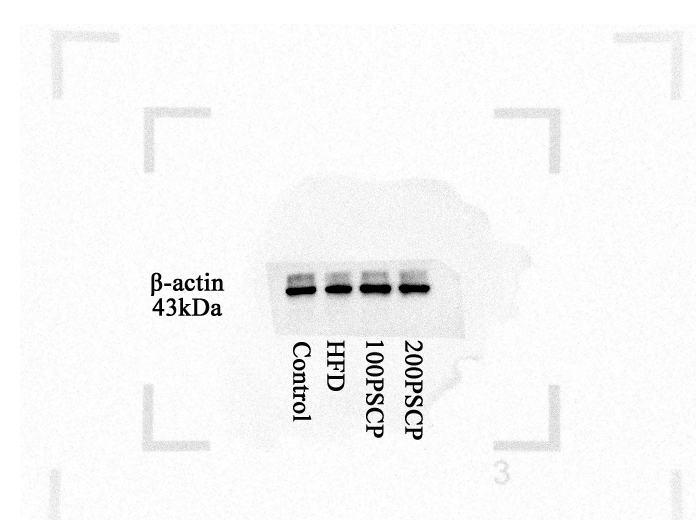

Supplement: Supplementary file 1 [file Data_Sheet_1.ZIP › Original data/Western blots-Fig6/a┬-actin/a┬-actin-Repeat 3.tif]

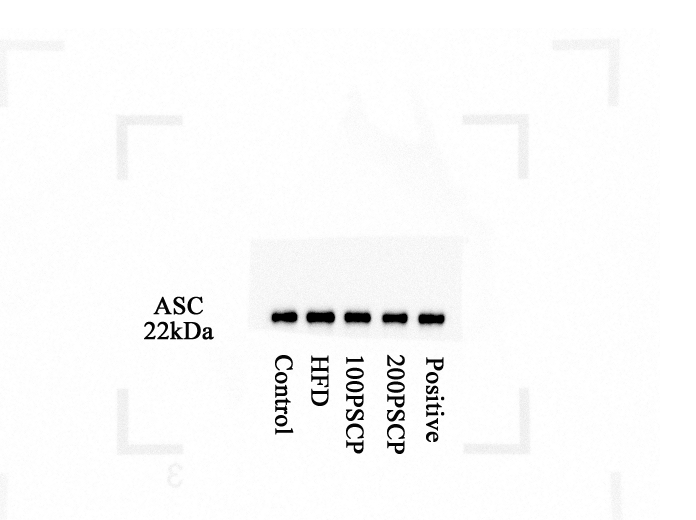

Supplement: Supplementary file 1 [file Data_Sheet_1.ZIP › Original data/Western blots-Fig7/ASC/ASC-Repeat 1.tif]

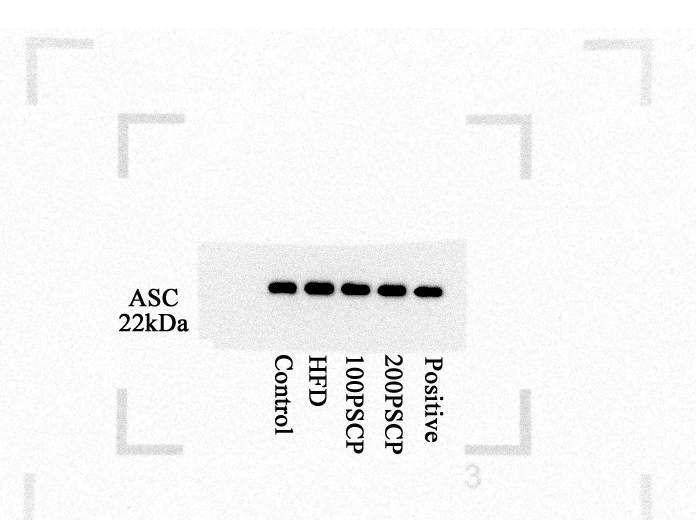

Supplement: Supplementary file 1 [file Data_Sheet_1.ZIP › Original data/Western blots-Fig7/ASC/ASC-Repeat 2.tif]

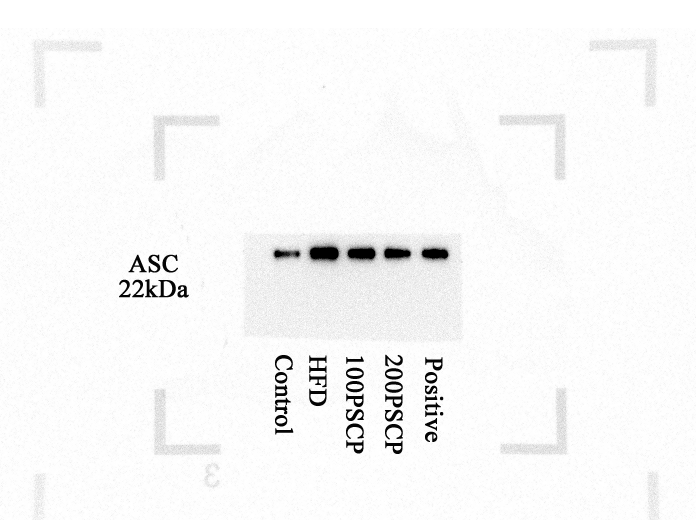

Supplement: Supplementary file 1 [file Data_Sheet_1.ZIP › Original data/Western blots-Fig7/ASC/ASC-Repeat 3.tif]

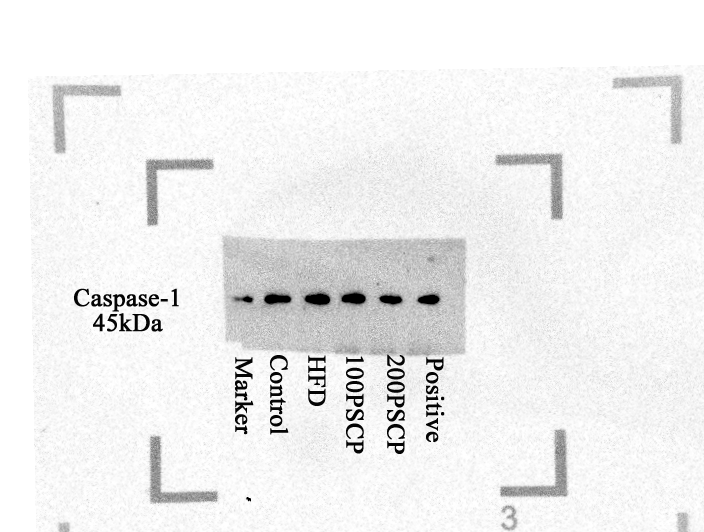

Supplement: Supplementary file 1 [file Data_Sheet_1.ZIP › Original data/Western blots-Fig7/Caspase-1/Caspase-1 -Repeat 1.tif]

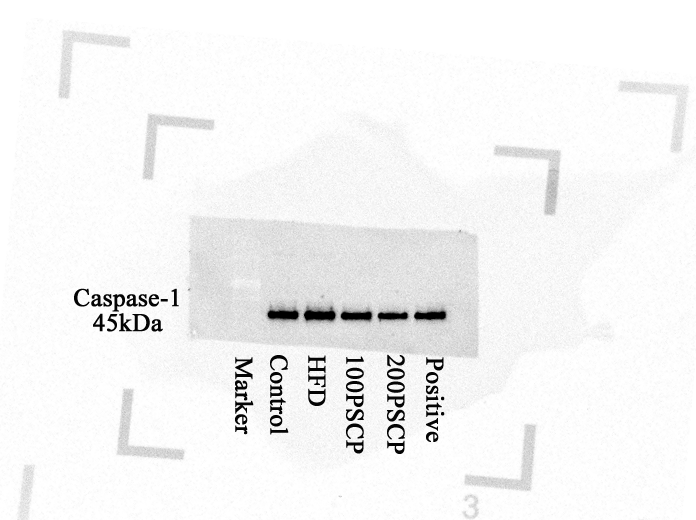

Supplement: Supplementary file 1 [file Data_Sheet_1.ZIP › Original data/Western blots-Fig7/Caspase-1/Caspase-1 -Repeat 2.tif]

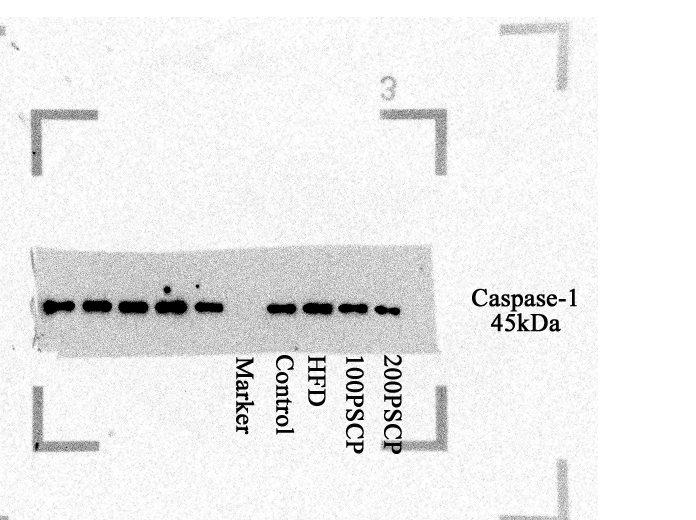

Supplement: Supplementary file 1 [file Data_Sheet_1.ZIP › Original data/Western blots-Fig7/Caspase-1/Caspase-1 -Repeat 3.tif]

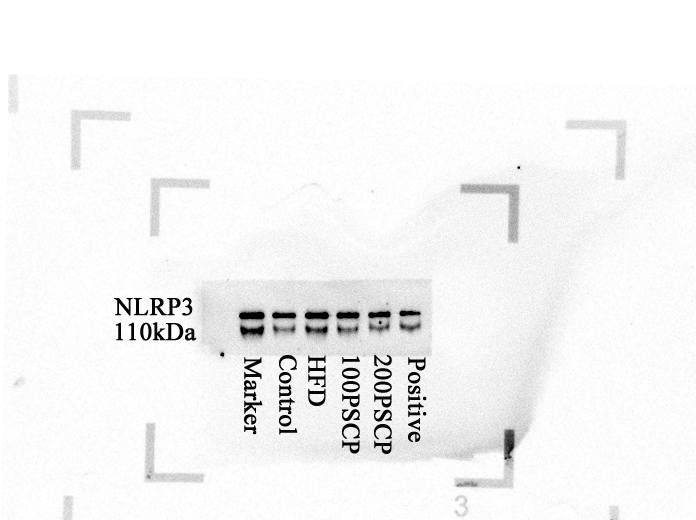

Supplement: Supplementary file 1 [file Data_Sheet_1.ZIP › Original data/Western blots-Fig7/NLRP3/NLRP3-Repeat 1.tif]

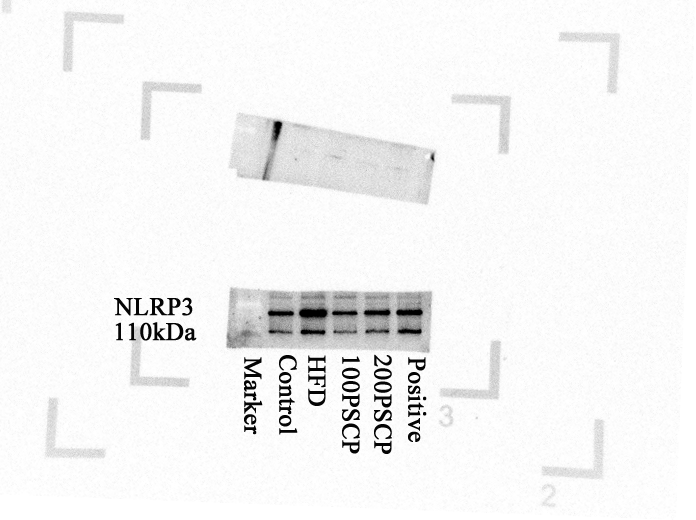

Supplement: Supplementary file 1 [file Data_Sheet_1.ZIP › Original data/Western blots-Fig7/NLRP3/NLRP3-Repeat 2.tif]

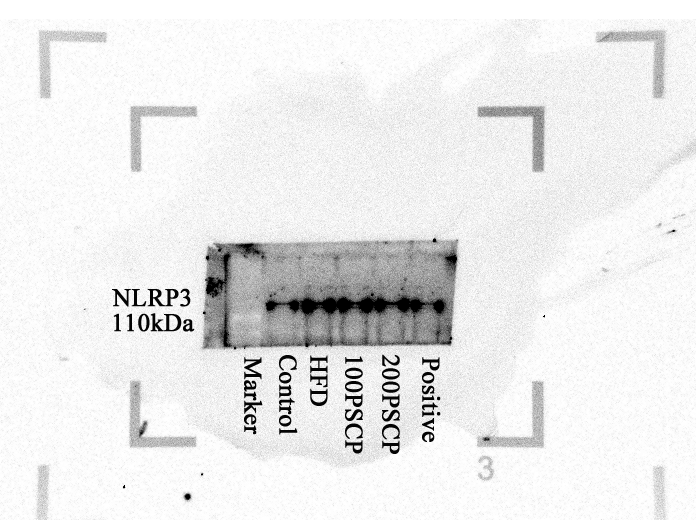

Supplement: Supplementary file 1 [file Data_Sheet_1.ZIP › Original data/Western blots-Fig7/NLRP3/NLRP3-Repeat 3.tif]

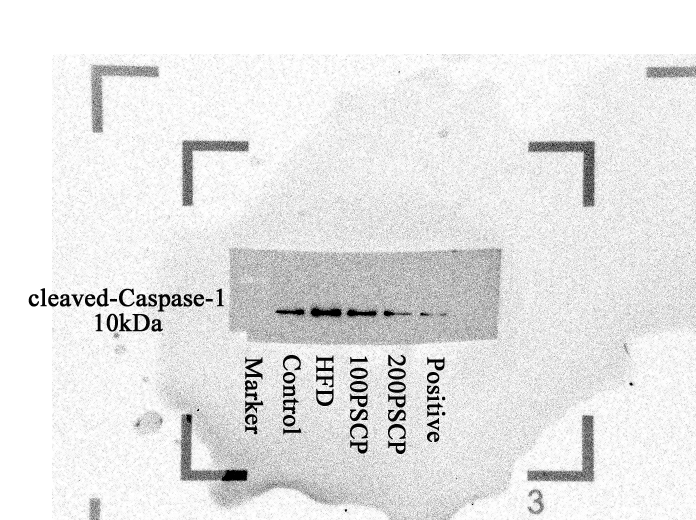

Supplement: Supplementary file 1 [file Data_Sheet_1.ZIP › Original data/Western blots-Fig7/cleaved-caspase-1/cleaved-Caspase-1 -Repeat 1.tif]

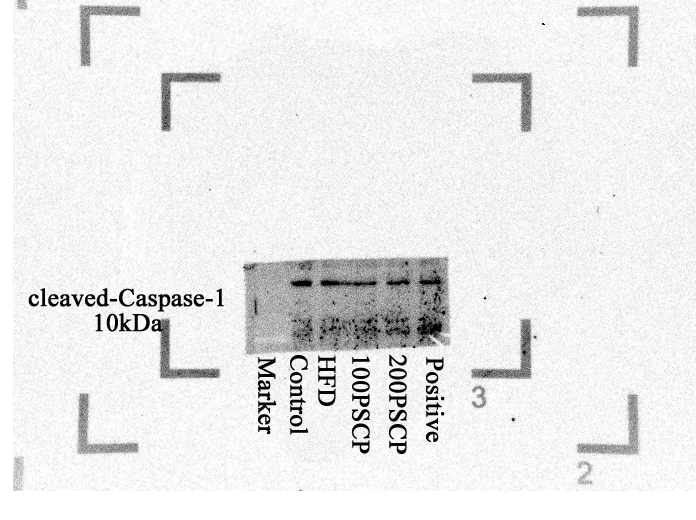

Supplement: Supplementary file 1 [file Data_Sheet_1.ZIP › Original data/Western blots-Fig7/cleaved-caspase-1/cleaved-Caspase-1 -Repeat 2.tif]

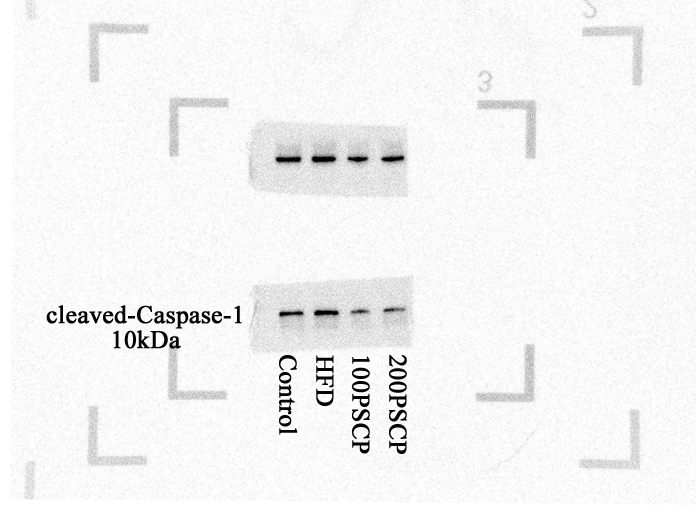

Supplement: Supplementary file 1 [file Data_Sheet_1.ZIP › Original data/Western blots-Fig7/cleaved-caspase-1/cleaved-Caspase-1 -Repeat 3.tif]

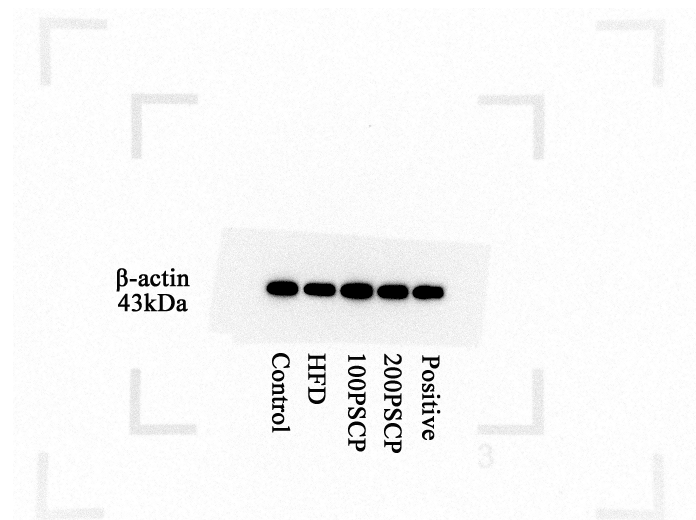

Supplement: Supplementary file 1 [file Data_Sheet_1.ZIP › Original data/Western blots-Fig7/a┬-actin/a┬-actin-Repeat 1.tif]

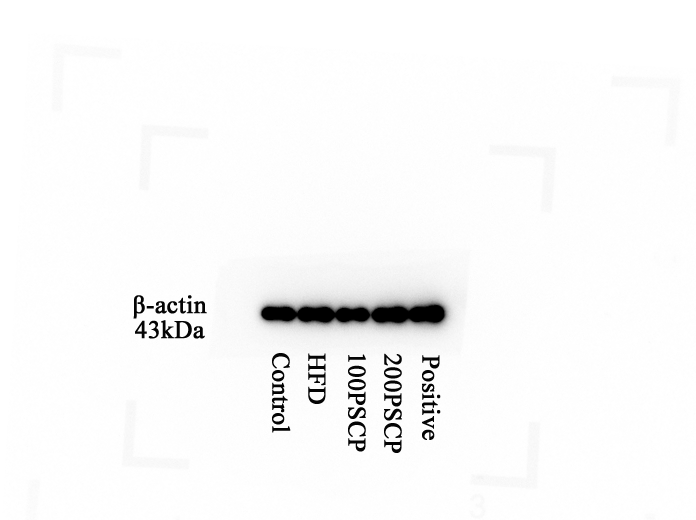

Supplement: Supplementary file 1 [file Data_Sheet_1.ZIP › Original data/Western blots-Fig7/a┬-actin/a┬-actin-Repeat 2.tif]

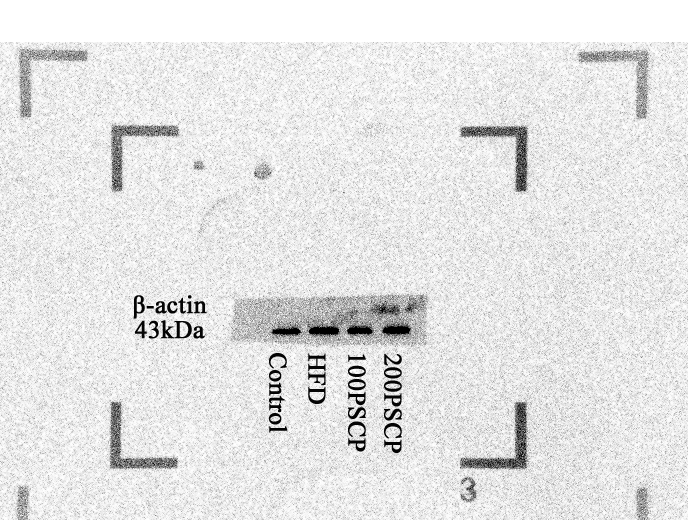

Supplement: Supplementary file 1 [file Data_Sheet_1.ZIP › Original data/Western blots-Fig7/a┬-actin/a┬-actin-Repeat 3.tif]
